# Unveiling nuclear chromatin distribution using IsoConcentraChromJ: A flourescence imaging plugin for IsoRegional and IsoVolumetric based ratios analysis

**DOI:** 10.1371/journal.pone.0305809

**Published:** 2024-07-02

**Authors:** Lama Zeaiter, Ali Dabbous, Francesca Baldini, Aldo Pagano, Paolo Bianchini, Laura Vergani, Alberto Diaspro

**Affiliations:** 1 Department for the Earth, Environment and Life Sciences, University of Genoa, Genova, Italy; 2 Nanoscopy, Istituto Italiano Tecnologia, Genoa, Italy; 3 Department of Electrical, Electronic and Telecommunication Engineering, University of Genoa, Genova, Italy; 4 Department of Experimental Medicine, University of Genoa, Genova, Italy; 5 IRCCS Ospedale Policlinico San Martino, Genova, Italy; 6 Department of Physics, University of Genoa, Genova, Italy; Universita degli Studi Gabriele d’Annunzio Chieti e Pescara, ITALY

## Abstract

Chromatin exhibits non-random distribution within the nucleus being arranged into discrete domains that are spatially organized throughout the nuclear space. Both the spatial distribution and structural rearrangement of chromatin domains in the nucleus depend on epigenetic modifications of DNA and/or histones and structural elements such as the nuclear envelope. These components collectively contribute to the organization and rearrangement of chromatin domains, thereby influencing genome architecture and functional regulation. This study develops an innovative, user-friendly, ImageJ-based plugin, called IsoConcentraChromJ, aimed quantitatively delineating the spatial distribution of chromatin regions in concentric patterns. The IsoConcentraChromJ can be applied to quantitative chromatin analysis in both two- and three-dimensional spaces. After DNA and histone staining with fluorescent probes, high-resolution images of nuclei have been obtained using advanced fluorescence microscopy approaches, including confocal and stimulated emission depletion (STED) microscopy. IsoConcentraChromJ workflow comprises the following sequential steps: nucleus segmentation, thresholding, masking, normalization, and trisection with specified ratios for either 2D or 3D acquisitions. The effectiveness of the IsoConcentraChromJ has been validated and demonstrated using experimental datasets consisting in nuclei images of pre-adipocytes and mature adipocytes, encompassing both 2D and 3D imaging. The outcomes allow to characterize the nuclear architecture by calculating the ratios between specific concentric nuclear areas/volumes of acetylated chromatin with respect to total acetylated chromatin and/or total DNA. The novel IsoConcentrapChromJ plugin could represent a valuable resource for researchers investigating the rearrangement of chromatin architecture driven by epigenetic mechanisms using nuclear images obtained by different fluorescence microscopy methods.

## Introduction

Advancements in optical microscopy instrumentation and labelling methods have enabled the visualization of biological macromolecules at near-atomic resolution directly within tissues, cells, organelles [1, 2]. However, analysis of these images poses significant challenges regarding accuracy and interpretation of data [3]. A critical point is the quantitative analysis of the distribution of specific macromolecules within the three-dimensional (3D) framework of cells or nuclei, and how this distribution may change in response to intrinsic or extrinsic stimuli [4]. Adipogenesis represents a complex biological process wherein pre-adipocytes undergo steatogenic differentiation into lipid-loaded mature adipocytes. During adipogenesis, a marked remodeling occurs under the control of a network of events involving signaling pathways, transcription factors, and gene expression changes. The in vitro differentiation of the 3T3-L1 fibroblast cells into mature adipocytes represents a reliable and widely employed model for structural and functional studies. Chromatin is a complex macromolecule resulting from the ordered assembly of DNA with histone and non-histone proteins and cohesins. In particular, the cohesin complex mediates sister chromatid cohesion. All together, these components regulate gene expression by organizing and stabilizing the chromatin structure thus guaranteeing the functional organization of the eukaryotic genome [5, 6]. Chromatin structure influences and regulates the DNA transcription, replication, and repair [7]. In the nucleus, chromatin is organized into distinct domains with distinct patterns of epigenetic modifications [8–11]. The interphase chromosomes, commonly called chromosomal territories, occupy distinct spatial regions within the nucleus [12]. These territories seem to adopt a spherical shape, ranging in diameter from 2 to 4 μm, with minimal overlapping with neighbor territories [12]. Euchromatin, characterized by heightened gene expression, tends to localize nearer to the center of the nucleus. In contrast, heterochromatin, which is less transcriptionally active, is positioned closer to the nuclear envelope. Additionally, highly transcribed regions are observed in close proximity to the nuclear pores, and this may facilitates the efficient mRNA. This spatial organization underscores the intricate relationship between chromatin architecture and functional genomic processes [13]. To unravel the changes in chromatin distribution during physiological processes a crucial step is to quantitatively analyze the chromatin distribution across the nucleus area/volume. Various algorithms with many configurations and platforms are already available [14, 15], but super-resolution optical microscopy requires the development of ad hoc image analysis tools to investigate the spatial organization and epigenetics of chromatin at unprecedented levels of detail [16]. Indeed, a lot of machine learning algorithms or plugins have been designed for the processing of 2D and 3D biological images within the ImageJ software platform [17, 18], but they have poor efficiency in accommodating different microscopy methods, scales, and experimental conditions. A high level of expertise is usually required to the user to choose the right algorithm, to fine-tune the parameters, to create customized models for each experiment, to prepare annotations, to train models and to configure algorithms [4, 19]. Moreover, classical algorithms may be unable to adapt to the heterogeneity of biological samples or to technical artifacts [20]. Therefore, novel plugins for optimizing the procedure of cell and/or nucleus segmentation become necessary [21]. In the present paper, we propose a novel, simple, user-friendly plugin that can be readily shared and easily applied across various fluorescence microscopy modalities without requiring additional user’s interventions. The plugin has been tested on nuclear images acquired by two optical nanoscale microscopies, the confocal and the stimulation emission depletion (STED) microscopy [22, 23], that provide high axial and lateral optical resolution allowing to appreciate the rearrangement of chromatin domains in both 2D and 3D spatial contexts. The workflow involves the following sequential steps: nuclei segmentation, appropriate thresholding, masking, edge detection, normalization, and area splitting for both 2D and 3D acquisitions. The plugin is embedded in a streamlined workflow, and extracts different concentric regional (2D) or volumetric (3D) ratios facilitating the quantitative analysis of fluorescence distribution in a concentric manner within 2D and 3D nuclear images. The plugin workflow has been tested on pre-adipocytes and mature adipocytes, allowing the discernment of distinct zones within the nucleus showing different chromatin compaction and epigenetics [24].

## Materials and methods

### Cell culture and staining

The pre-adipocyte cell line 3T3L1, mouse fibroblasts, is supplied by ATCC (American Type Culture Collection, Manassas, VA, USA). To induce adipogenic differentiation, confluent pre-adipocytes cultured in DMEM medium were exposed to adipogenic mixture consisting of 1.7 μM insulin, 1 μM dexamethasone (DEXA), and 500 μM 3-isobutyl-1-methylxanthine (IBMX) for 48 hours [25]. Subsequently, the medium was replaced with fresh medium containing 1.7 μM insulin, and medium changes were performed every 48 hours for a total of 6 days [26, 27]. On the 7th day, the cells were harvested for microscopy acquisition and seeded on collagenated glass coverslides. After fixation and permeabilization, both pre-adipocytes and mature adipocytes were immunostained to visualize the acetylated histone, H3K9Ac using a primary rabbit antibody (Invitrogen, MA5–11195; 41 μg/mL, 1:200) at 4 °C, and a secondary anti-rabbit antibody (Atto 647 N Sigma 49839; 1mg/ml, 1:200) at room temperature. Both antibodies were employed at a dilution of 1:200, following the manufacturer’s instructions. The nuclear DNA was stained with either Hoechst 33342 dye (3.24μM; Invitrogen, Thermo-Fisher, Massachusetts, USA) for 10 minutes to obtain 3D acquisitions by Confocal, and Syto13 dye (2μM; Invitrogen, Thermo-fisher Scientific) for 25 minutes to obtain 2D acquisitions by STED.

### Fluorescense microscopy

3D imaging was conducted using a Nikon A1R MP confocal microscope equipped with a Nikon Plan Apo VC 100×/1.40 oil immersion objective lens (Nikon Instruments, Tokyo, Japan). For 2D analysis, a Leica Stellaris 8 Tau-STED microscope with an HC PL APO CS2 100×/1.40 oil immersion objective lens (Leica Microsystems, Mannheim, Germany) was employed [28]. This microscope includes a donut-shaped STED laser beam operating at =775 nm. For each cell condition, pre-adipocytes and mature adipocytes, 10 images were analyzed for 2D acquisitions and 10 images for 3D acquisitions. Consistent imaging was maintained at a field of view of 71.68 x 71.68 microns (1024x1024) with pixel size 0.07 x 0.07 microns. Following image acquisition, subsequent processing employed the ImageJ software, utilizing the plugin to adhere to a prescribed workflow tailored for chromatin analysis [25].

## Results

### IsoConcentraChromJ plugin

**Fig 1:**
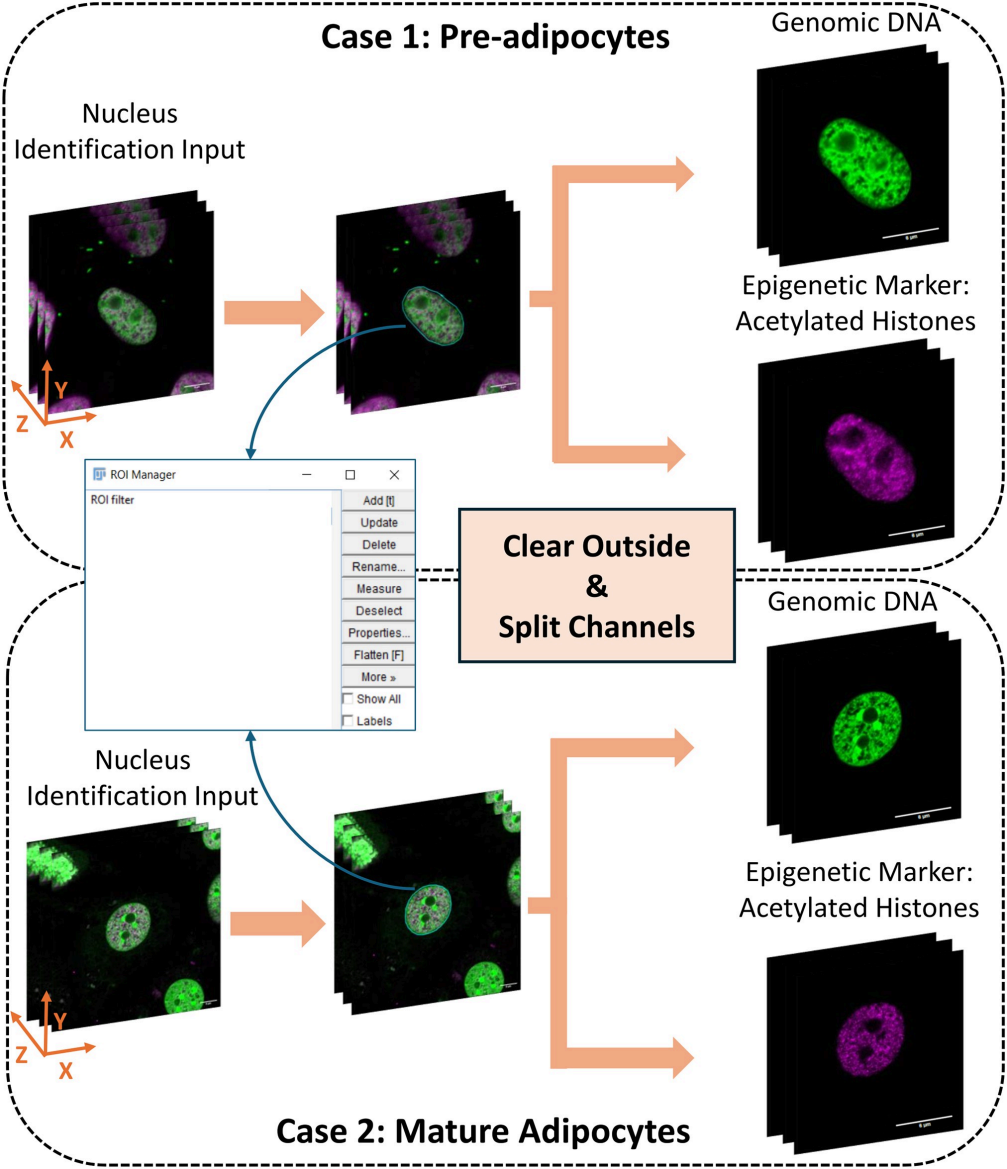
Summary of the visualizing and processing images for a representative nucleus of both pre-adipocytes and mature adipocytes. Input Image (left), Sequential ROI Filtering (middle), and Splitting Channels (right) for Genomic DNA and Epigenetic Marker (Acetylated Histones) in 3D Imaging. Scale bar: 6μm.

**Fig 2:**
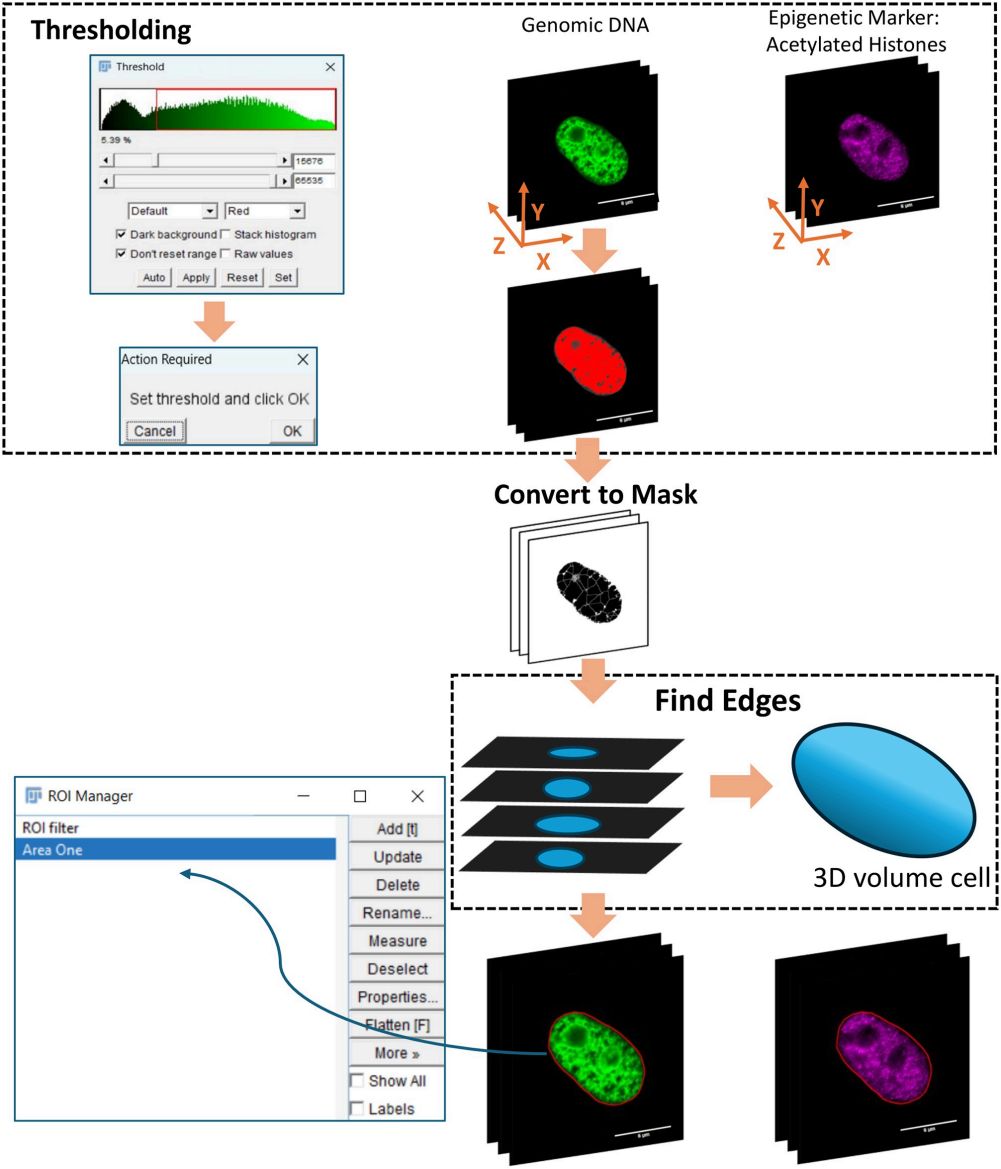
Filtering procedure for a pre-adipocyte nucleus. Detection of Genomic DNA (Channel 1) with Synchronous Edge Selection on Epigenetic Acetylated Histones Marker (Channel 2). Scale bar: 6μm.

**Fig 3:**
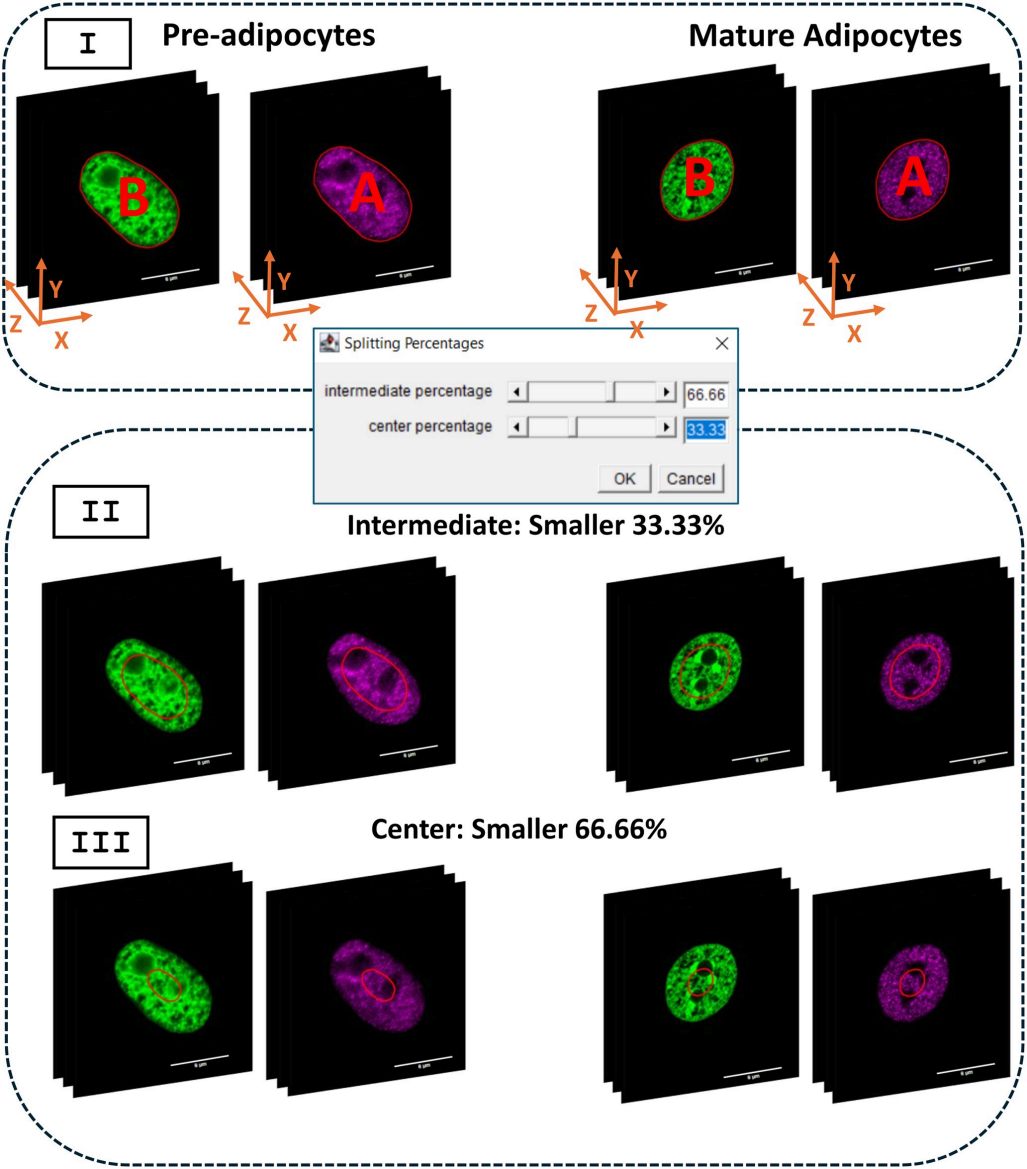
Area regions. Visualization of the area regions; Representative Areas Highlighted in Red Bold for Full, Intermediate, and Center Regions. (Top) Full Area (I) of two channels, Genomic DNA and Epigenetic Marker (Acetylated Histones) respectively. (Middle) splitting percentage dialog for selecting the percentage of splitting for the intermediate and center area. (Bottom) intermediate area of the two channels selected using imageJ Plugin and it represents 66.66% of the full area (II) and center area, where it represents 33.33% of the full area (III) selected using the ImageJ plugin. Scale bar: 6μm.

The IsoConcentraChromJ plugin follows a structured workflow consisting of four key mechanisms, each applied sequentially for user convenience by one click. These mechanisms are designed to streamline the analysis of nuclear images, as illustrated in S1 Fig. A breakdown of each step is summarized as follows: (i) “Clear Outside and Image Splitting” where relevant objects within the nucleus image are selected, and splitting the input identified nucleus into channels is provided (Fig 1); (ii) “Nucleus Filtering and Edge Detection” where nucleus can be selected based on physical features and signal intensity, precisely delineating the boundaries of the nucleus (Fig 2); (iii) “Nucleus Splitting” where the identified nucleus boundary is divided into distinct regions (Fig 3); (iv) “Statistical Analysis” where further computations and measurements are applied. The analytical process differs between 2D and 3D images. In the case of 2D images, intensity calculations are executed on a single slice, whereas for 3D images, intensity is calculated across the entire z-stack. The plugin is then employed to normalize the intensity levels in the measurements of concentric ratios for the nucleus. This structured approach ensures that users can efficiently process both 2D and 3D images while maintaining the flexibility to customize their analysis based on specific research needs.

#### Clear outside and image splitting

This step is a pivotal starting point within our plugin’s workflow. Thanks to ImageJ plugins (“Clear Outside” and “Split Channels”), that makes our implementation straight forward. This methodology begins by manually selecting a nucleus of interest among many nuclei visible within the image. Then, IsoConcentraChromJ is applied to clear the area outside the selected nucleus and to break down the input identification into two individual channels: Genomic DNA and Epigenetic Marker, acetylated histones in our test. This module operates by removing any unwanted elements that lie outside the user-defined region of interest before executing the ensued steps of the plugin. This crucial step ensures that all ensuing analyses exclusively revolves around the pertinent components within the nucleus, a visual representation of which can be seen in Fig 1. What makes this mechanism particularly powerful is its ability to simplify the image by splitting it into its fundamental components of our interest. This splitting facilitates the analysis and independent processing of different channels, which is especially valuable for researchers dealing with multi-channel or multi-modal images in the ImageJ environment. Essentially, the “Clear Outside and Image Splitting” mechanism acts as a streamlined gateway into the image-processing pipeline, enhancing the precision and efficiency of the analysis of cellular structures and components found within the image.

#### Nucleus filtering

To identify the nuclei of interest within the image, our plugin allows a sequential procedure with a series of steps, including thresholding and masking. The threshold dialog is summarized in Fig 2-left: here, the user has the flexibility to choose one of the ImageJ threshold algorithms from a dropdown list right within the dialog. Furthermore, users have the option to fine-tune the thresholding process by adjusting the minimum and maximum intensities. Users can either work with the default thresholding settings or manually configure the thresholding mechanism to suit their specific needs. Once the configuration is complete, users need to confirm their choices to apply the thresholding to either the entire z-stack in the case of 3D images or to a single stack in 2D images. This approach empowers users to tailor the thresholding process according to their image data and research objectives, ensuring precise and customised results. In the present study, the default threshold algorithm was employed for all the image acquisition either 2D or 3D, as shown in Fig 2. After thresholding, we perform masking to facilitate the easy identification of the boundary edges of the nucleus. This masking step is a valuable addition as it simplifies the process of isolating and highlighting the edges for further analysis, which in our case is the nucleus of the adipocytes. To uncover these boundary edges, our plugin navigates through all the z-stacks, identifying the edges within each stack (Fig 2). The thresholding and masking procedures were implemented in our plugin using imageJ built in functions (“Threshold…” and “Convert to Mask”). By the end of this process, that is aiming to study the concentric distributions of epigenetic modifications, one of the main contributors of chromatin arrangement, the plugin successfully pinpoints and outlines the boundaries of the nucleus, and “Area One” region of interest (ROI) is drawn over the nucleus and saved in ROI Manager for later use as shown in the right of Fig 2.

#### Nucleus splitting

Our plugin draws inspiration from the study of concentric remodeling and introduces a novel mechanism called “nucleus splitting”. This mechanism is designed to perform a partition of the nucleus into multiple distinct regions based on biological characteristics. To achieve this, we employ thresholding and masking techniques to define the nucleus’s full area as above described. Specifically, our plugin (Fig 3) started with splitting the channels referring to two fluorescent labeling: Channel one, represents to the genomic DNA, denoted as “B”, and channel two, representing an epigenetic marker: acetylated histone in our case, denoted as “A”. Each channel was further divided into three distinct regions each: (i) “Full Nucleus Region”; (ii) “Intermediate Area”; and (iii) “Center Area”. The “Intermediate Area” is defined as a region that reduces the full area by 33.33%, while the “Center Area” is a region that reduces the full area by 66.66% (Fig 3). For the practical implementation of this division in ImageJ software, we make use of convenient functions, such as “get Selection Coordinates” and “make Selection” which can be easily incorporated into our plugin code to execute this task. The new region’s coordinates are calculated using the following equations:

$$\begin{array}{c} x_{\text{new}}=\left(X_{\text{Full}}-\text{Xcentroid}\right)*\left({\text{Shrink}}_{\text{Factor}}+\text{Xcentroid}\right) \end{array}$$ (1)

$$\begin{array}{c} y_{\text{new}}=\left(Y_{\text{Full}}-\text{Ycentroid}\right)*\left({\text{Shrink}}_{\text{Factor}}+\text{Ycentroid}\right) \end{array}$$ (2)

The variables *x*_*new*_ and *y*_*new*_ denote the updated coordinates for the newly defined regions. These coordinates are determined by applying a *Shrink*_*Factor*_, which signifies the extent of reduction. In our study, the *Shrink*_*Factor*_ is set at 33.33% for the intermediate-sized regions and 66.66% for the center regions. The reference points for these calculations are the centroid coordinates, “X” and “Y”, which represent the center of the full area. Once these new coordinates have been computed using our code in conjunction with the implemented ImageJ functions, we are able to generate fresh selections and store them within the ROI Manager Dialog as shown in Fig 3. This design allows users to conveniently adjust the number of regions they wish to analyze by simply modifying or adding new shrink factors. In essence, this system grants users the flexibility to customize the division of regions as per their specific needs by manipulating the shrink factors.

#### Statistical analysis

As last step, our plugin proceeds to assess further details, focusing on the examination of concentric region characteristics. This approach revolves around the calculation of concentric ratios, with a focus on specific areas located within the nucleus and distributed across the three regions defined earlier. In particular, we identify and delineate specific areas of interest within the two channels, namely genomic DNA and the epigenetic marker. These specific areas serve as the basis for the subsequent analysis, enabling us to investigate and calculate concentric ratios within the nucleus. In this step, our primary objective is to compute the integrated density for each of the specified areas and subsequently determine the concentric ratios. Before delving into this analysis, a crucial preliminary step is the normalization. For normalization, the user must draw a Region of Interest (ROI) that serves as the background subtraction area within the two previously mentioned channels. This ROI is saved manually to the ROI Manager dialog for future reference. Then, the plugin automatically selects the background ROI for normalization and calculates the Normalization Factor using the following equation: Scale bar: 6μm.


$$\begin{array}{c} {\text{NF}}_{\text{DNA}}=\left(\frac{\text{total}\_\text{MID}\_\text{DNA}}{nSlices}\right)*\left(\text{voxel}\_\text{Depth}\right) \end{array}$$ (3)

$$\begin{array}{c} {\text{NF}}_{\text{Acetylated}}=\left(\frac{\text{total}\_\text{MID}\_\text{Acetylated}}{nSlices}\right)*\left(\text{voxel}\_\text{Depth}\right) \end{array}$$ (4)

The two parameters *NF*_*DNA*_ and *NF*_*Acetylated*_ represent the normalization factors for the single channels (DNA and Acetylation). These factors allow to standardize the data across different regions of interest, and their calculation uses the Mean Integrated Density (MID) of the background subtraction area MID. MID is derived from the z-stacks for 3D acquisitions, or a single plane in 2D acquisitions. The MID can be collected from the plugin using a function called getResults(“Mean”). In the 3D context, the normalization factor is adjusted by multiplying it with the depth of the voxel, while in the 2D context, we operate with pixels and do not need to consider voxel depth. This adjustment ensures that the normalization factors are appropriately applied, accounting for the specific dimensional context of the analysis. Normalization is fundamental to correct for differences in the background intensity, providing more accurate determinations. As reported in S1 Fig, the specific areas are denoted as B1, B2, and B3 (for the Genomic DNA, channel B) and A1, A2, and A3 (for the epigenetic marker, channel A). The following step moved to calculate the Integrated Density (IntDen) within each of the segmented areas, encompassing the full, medium, and small regions. The MID can be collected from the plugin using a function called getResults(“IntDen”). For 3D data, we account for the volumetric aspect by multiplying the IntDen value with the depth. To normalize the total IntDen for each area, we employ the following equation:

$$\begin{array}{c} {\text{Normalized}}_{{\text{Intensity}}_{\text{area}}}={\text{IntDen}}_{\text{area}}-\left(NF*area\right) \end{array}$$ (5)

This normalization procedure is implemented to correct variations in the background level, ensuring that the total IntDen values are appropriately adjusted, thus making our analyses more reliable. In our pilot study, we calculated ten ratios, denoted as A/B, A1/B, A1/A, A2/B, A2/A, A3/B, A3/A, B1/B, B2/B, and B3/B. For example, the value. A1 is determined by computing the disparity between the Integrated Density of the entire region in the Acetylated channel and that of the intermediate region within the same Acetylated channel. These ratios allow us to explore various aspects of the biological data, comparing and contrasting different regions and channels.

#### 2D and 3D biological analyses

**Fig 4:**
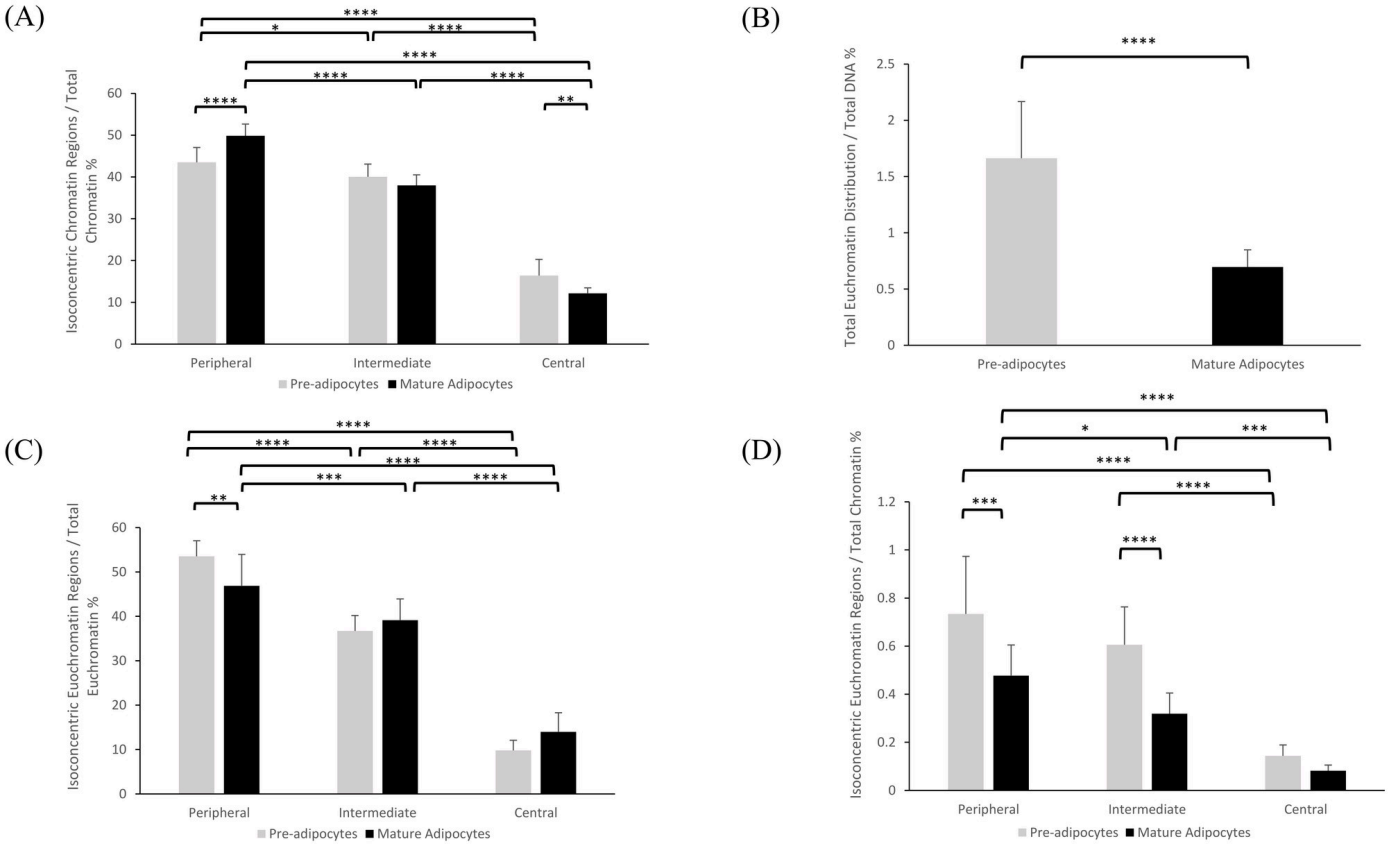
2D spatial distribution of total chromatin and euchromatin in pre-adipocytes and mature adipocytes. The ratios represented in percentages. (A) B1/B, B2/B, and B3/B Ratios: Chromatin organization across peripheral, intermediate, and central regions within the nucleus relative to total chromatin; (B) A/B Ratio: Total euchromatin relative to total chromatin in Pre-adipocytes and Mature Adipocytes; (C) A1/B, A2/B, and A3/B Ratios: H3K9Ac-associated euchromatin in Pre-adipocytes and Mature Adipocytes Across Nuclear Chromatin Regions with respect to total chromatin; (D) Isoconcentric Distribution of euchromatin Regions Relative to Total euchromatin Within Nuclei, A1/A, A2/A, and A3/A Ratios: Isoconcentric organization of euchromatin across the peripheral, intermediate, and central nuclear regions, respectively, in Pre-adipocytes and Mature Adipocytes.

**Fig 5:**
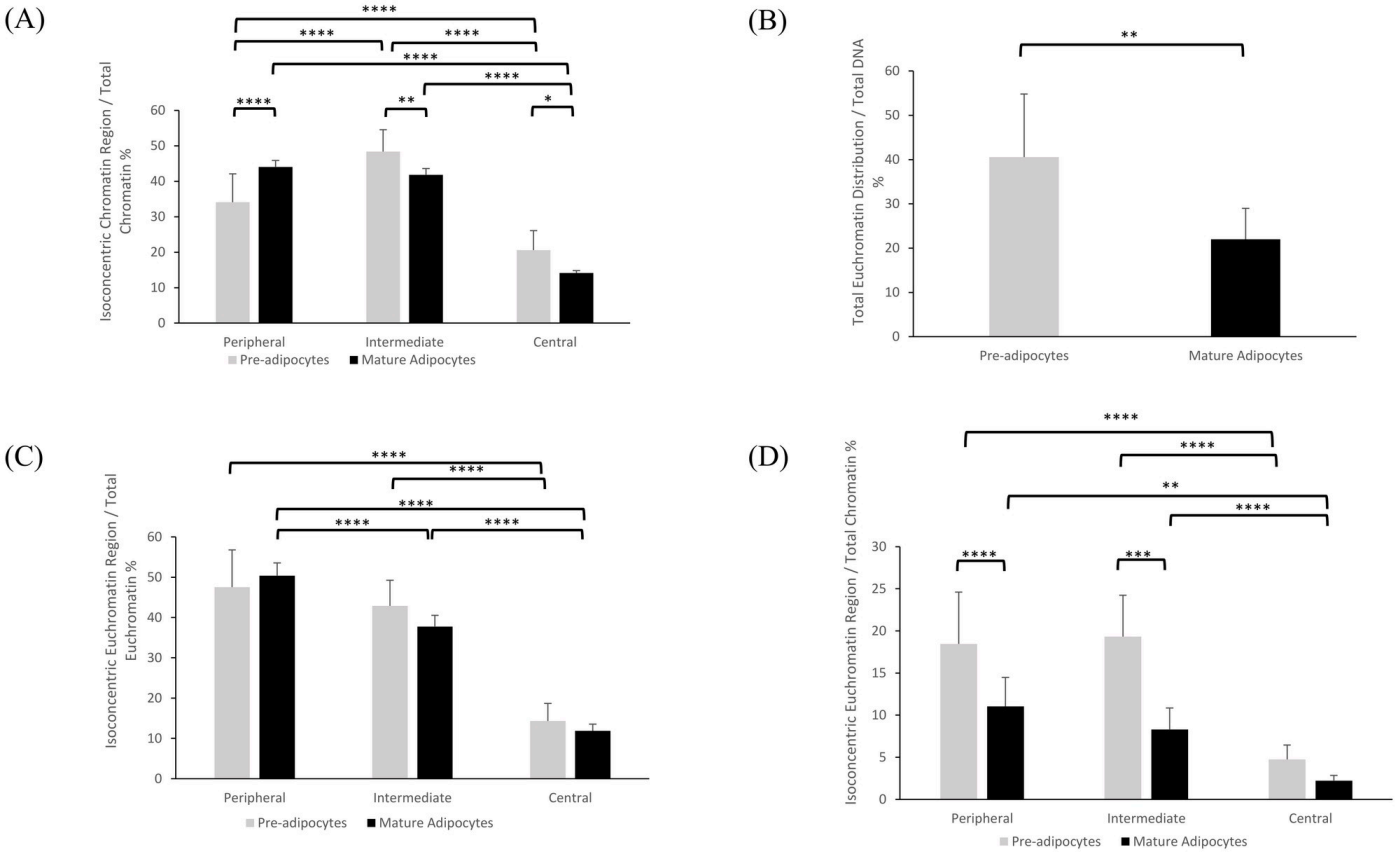
3D spatial organization of chromatin and euchromatin dynamics during adipocyte maturation. The ratios represented in percentages. (A) B1/B, B2/B, and B3/B Ratios: Chromatin organization across peripheral, intermediate, and central regions within the nucleus relative to total chromatin; (B) A/B Ratio: Total euchromatin relative to total chromatin in Pre-adipocytes and Mature Adipocytes; (C) A1/B, A2/B, and A3/B Ratios: H3K9Ac-associated euchromatin in Pre-adipocytes and Mature Adipocytes Across Nuclear Chromatin Regions with respect to total chromatin; (D) Isoconcentric Distribution of euchromatin Regions Relative to Total euchromatin Within Nuclei, A1/A, A2/A, and A3/A Ratios: Isoconcentric organization of euchromatin across the peripheral, intermediate, and central nuclear regions, respectively, in Pre-adipocytes and Mature Adipocytes.

A comprehensive evaluation of IsoConcentraChromJ was carried on by using 2D and 3D images acquired by fluorescence microscopy. By leveraging our plugin, a series of ratios calculated on concentric regions of the nuclei are obtained that allows us to assess the spatial organization/distribution of the chromatin Figs 4 and 5). Fig 4A illustrates the changes in 2D concentric chromatin distribution from pre-adipocytes to mature adipocytes, considering both total chromatin and acetylated chromatin (H3K9 acetylation). Three ratios (B1/B, B2/B, and B3/B) have been calculated for total chromatin to quantitatively assess its distribution across the peripheral, intermediate, and central regions of the nucleus, respectively. In mature adipocytes, we observed an increase in the B1/B ratio compared to pre-adipocytes (43.53 ± 3.4 vs. 49.87± 2.80; p≤0.0001), suggesting a potential diffusion of chromatin towards the periphery during adipocyte maturation. No statistically significant difference was detected for the chromatin distribution in the intermediate region (B2/B ratios) between pre-adipocytes and mature adipocytes. Conversely, the B3/B ratios revealed a significant decrease from pre- to mature adipocytes (16.40 ± 3.90 vs 12.15 ± 1.31; p≤0.01) thus confirming that chromatin is more concentrated in the central region of the nuclei in pre-adipocytes respect to mature adipocytes. Of note, in both pre-adipocytes and mature adipocytes we observed a reduced content of chromatin in the central region. Moreover, pre-adipocytes showed significant differences in chromatin distribution between peripheral and intermediate regions (p≤0.01), peripheral and central regions (p ≤ 0.0001), and intermediate and central regions (p≤0.0001). Similar results were obtained in mature adipocytes, where differences persist between peripheral and intermediate regions (p≤0.0001), peripheral and central regions (p≤0.0001), and intermediate and central regions (p≤0.0001). Then, we assessed the distribution of acetylated chromatin within the nucleus, specifically focusing on H3K9 acetylation as a marker for euchromatin. Firstly, the A/B ratio was employed to gauge the proportion of H3K9Ac-euchromatin relative to total chromatin Fig 4B. In pre-adipocytes, this ratio was significantly higher compared to mature adipocytes (1.7 ± 0.50 vs 0.7 ± 0.15; p≤0.0001), indicative of a more globally active chromatin state in pre-adipocytes. To explore the concentric distribution of H3K9Ac-euchromatin across nuclear regions, three ratios (A1/B, A2/B, and A3/B) were utilized Fig 4C. In pre-adipocytes the A1/B ratio was higher than in mature adipocytes (0.7 ± 0.24 vs 0.5 ± 0.13; p ≤ 0.001), suggesting a prevalence of euchromatin in the peripheral region of pre-adipocytes. Also the A2/B ratio was higher in pre-adipocytes compared to mature adipocytes (0.61 ±0.16 vs 0.32 ± 0.09; p≤0.0001), whereas no statistically significant difference was observed between pre-adipocytes and mature adipocytes in the central regions (A3). Moreover, pre-adipocytes, exhibit significant differences among the peripheral (A1/B) and intermediate (A2/B) regions (p≤ 0.001), peripheral (A1/B) and central (A3/B) regions (p≤0.0001), and intermediate (A2/B) and central (A3/B) regions (p≤0.0001). Similarly, in mature adipocytes, notable differences were observed for the peripheral (A1/B) versus intermediate (A2/B) (p ≤ 0.05), peripheral (A1/B) versus central (A3/B) (p ≤ 0.0001), and intermediate (A2/B) versus central (A3/B) (p≤0.001) regions. Therefore, IsoConcentraChromJ is able to discriminate and quantify the spatially heterogeneous organization of chromatin and euchromatin within the nucleus, with distinct patterns observed between pre-adipocytes and mature adipocytes. We investigated also the isoconcentric distribution of euchromatin regions relative to the total euchromatin (Fig 4D). In pre-adipocytes, a significantly higher A1/A ratio was observed compared to mature adipocytes (53.51 ± 3.54 vs 46.89 ± 7.08; p≤0.01), indicating a more pronounced enrichment of euchromatin in the peripheral region of pre-adipocytes. Conversely, both the A2/A and A3/A ratios did not exhibit significant differences between pre-adipocytes and mature adipocytes. In pre-adipocytes, significant differences were observed in comparisons between peripheral (A1/A) and intermediate (A2/A) regions, peripheral (A1/A) and central (A3/A) regions, as well as intermediate (A2/A) and central (A3/A) regions, with all yielding p-values ≤ 0.0001. Similarly, in mature adipocytes, notable distinctions were evident, particularly in comparisons of peripheral (A1/A) versus intermediate (A2/A) regions (p ≤ 0.001), peripheral (A1/A) versus central (A3/A) regions (p ≤ 0.0001), and intermediate (A2/A) versus central (A3/A) regions (p ≤ 0.0001). In parallel with the 2D analysis, also the 3D concentric chromatin distribution was assessed considering both total chromatin and acetylated chromatin (H3K9) within the nucleus 3D framework (Fig 5A). Mature adipocytes display B1/B ratios higher than the pre-adipocytes 44.041 ± 1.85 vs. 34.08 ± 8.01 respectively, p≤0.0001), while pre-adipocytes show B2/B ratios higher than mature adipocytes (48.36 ± 6.17 vs. 41.83 ± 1.76, respectively, p≤ 0.01). as well as for the B3/B ratios s (20.581 ± 5.50 vs. 14.126 ± 0.67; respectively, p≤0.05), suggesting increased chromatin compaction toward the periphery in mature adipocytes and a preference toward the central nuclear area in pre-adipocytes, consistent with the 2D analyses.

In line with our 2D analysis, we aimed to thoroughly assess the 3D distribution of H3K9Ac-chromatin (euchromatin) within the nucleus, by using the A/B Fig 5B, A1/B, A2/B, and A3/B ratios Fig 5C. Our results unveiled a notably higher ratio of euchromatin to total chromatin in pre-adipocytes compared to mature adipocytes. Specifically, the A/B ratio is higher in pre-adipocytes then in mature adipocytes (40.566 ± 14.25 vs 21.992 ± 6.98; p≤0.01). Moreover, pre-adipocytes displayed significantly higher A1/B ratios compared to mature adipocytes (18.4636 ± 6.14 vs. 11.0373 ± 3.43 respectively, p≤0.001), and higher A2/B ratio compared to mature adipocytes 19.31 ± 4.91 vs. 8.28 ±2.56 respectively, p ≤ 0.0001). Furthermore, both pre-adipocytes and mature adipocytes exhibited a consistent pattern of decreasing chromatin distribution from the periphery towards the central region. Significant differences were notably observed within pre-adipocytes in comparisons between peripheral and central regions (p ≤ 0.0001), as well as intermediate and central regions (p ≤ 0.0001). Similarly, in mature adipocytes, significant disparities were evident in comparisons between peripheral and central regions (p ≤ 0.0001), and intermediate and central regions (p ≤ 0.01), emphasizing the significance of regional heterogeneity in elucidating chromatin organization within adipocytes. When we investigated the isoconcentric distribution of H3K9Ac relative to the total H3K9Ac content Fig 5D, we observed that mature adipocytes exhibit higher levels of H3K9Ac in the peripheral region, as indicated by the A1/A ratios (50.38 ± 3.16 vs. 47.52 ± 9.25; p ≤ 0.5521), that was balanced by the decrease in the A2/A and A3/A ratios compared to mature adipocytes (37.73 ± 0.27 vs. 42.88 ± 6.38; p ≤ 0.089; 11.18 ± 1.64 vs. 14.32 ± 4.33; p ≤ 0.6905). This suggests a preferential localization of euchromatin in the nuclear periphery of mature adipocytes. However, we can observe that both pre-adipocytes and mature adipocytes exhibited a gradual decrease in chromatin content from the peripheral to central regions of the nucleus. In pre-adipocytes, differences were present between peripheral vs. central region and intermediate vs. central regions (p ≤ 0.1355, p ≤ 0.0001, and p ≤ 0.0001, respectively). Similarly, mature adipocytes displayed significant differences among all pairs (p ≤ 0.0001, p ≤ 0.0001, and p ≤ 0.0001).

## Discussion and conclusion

Despite numerous studies have delved into investigating nuclear morphology and organization, our focus lied on studying the chromatin distribution within the 2D and 3D nucleus in quantitative terms during physiological processes such as adipogenesis. To this aim, we developed a novel ImageJ-based plugin (IsoConcentraChromJ) for qualitative and quantitative analysis of nuclear organization, tailored for studying epigenetic and structural chromatin patterns within the nucleus. The novel plugin allows us to detect and quantify alterations in chromatin distribution patterns during adipogenesis. Moreover, our results show that chromatin domains are not randomly distributed within the nucleus but have specific spatial patterns, potentially linked to functional roles, that represent different levels of chromatin compaction and transcriptional activity emphasizing the heterogeneity of nuclear structure, with implications for gene regulation and epigenetic processes. The primary advantage of our plugin lies in its ability to assess nuclear architecture by examining the distribution of epigenetic features and chromatin content using in vitro cellular models of pre- and mature adipocytes, utilizing both 2D and 3D imaging modalities. This is achieved through the calculation of ratios between specific concentric nuclear areas/volumes of the acetylated chromatin relative to total acetylated chromatin and/or total DNA. ImageJ software platform plugins handle the growing complexity of imaging datasets. In this study, we present an innovative image analysis tool developed on the ImageJ platform. This tool automates the segmentation of subnuclear chromatin domains by employing concentric splitting-data processing using both 2D and 3D fluorescence microscopy images. In our opinion, IsoConcentraChromJ offers a novel approach for investigating chromatin arrangements in both 2D and 3D images. The plugin has been tested to study adipogenesis and supplied some interesting biological results in terms of spatial localization of total chromatin and epigenetic domains between pre-adipocytes and mature adipocytes. As our knowledge, there is no imageJ plugin developed for nucleus area/volume based segmentation. Cunea et al. [29] developed an automatic tool to quantify chromatin compaction from 3D fluorescent images. While they focused on measuring numerous morphological parameters using single-channel and solely 3D images, our plugin introduces a significant novelty by utilizing multichannel images to conduct comparable ratio analysis related to the distribution of euchromatin and chromatin content. Poulet et al. developed the NucleusJ plugin to characterize 3D nuclear morphology and chromatin organization [30]. However, the analysis of the 77 nuclei took about 1 h, while our plugin takes only 2 seconds/ image. Dubos et al. developed the NodeJ for 3D segmentation of nuclear objects [31], that utilizes Laplacian algorithm, for automated object segmentation based on voxel intensity distribution in images and computed thresholds for nucleus segmentation, a more complex process than the default thresholding algorithm used. Our goal is to split the segmented nucleus in areas, which the NodeJ plugin does not support. Therefore, these three plugins have been developed for 3D image analysis purposes, while we are interested in both 2D and 3D image analysis, and for this reason our plugin supports both types of dimensionality. To this regard, our plugin represents the automation of a concentric analysis, eliminating the need for user intervention except for configuring an optimal threshold algorithm and splitting percentage parameters. The existing algorithms for edge detection often encounter challenges in accurately identifying the features mapping. In this context, our approach aims to enhance the precision of feature mapping, and to go beyond conventional methods, as it relies on the detection of pixel intensity and pattern recognition [32]. Moreover, a normalization methodology based on the mean background intensities is implemented in the plugin. Regarding the biological results, the iso-concentric analysis carried on by IsoConcentraChromJ divided the nucleus into regions of equal areas and volumes and provided interesting results on the distribution of chromatin and euchromatin across the nuclear substructure of adipocytes in different steps of maturation. The results showed a trend where chromatin diffuses towards the periphery during adipocyte maturation, while in pre-adipocytes chromatin is preferentially localized in the intermediate and central regions. Focusing on acetylated chromatin domains (euchromatin), we found that mature adipocytes show a reduction in euchromatin content compared to pre-adipocytes, suggesting dynamic shifts toward heterochromatin throughout adipogenesis. Additionally, isoconcentric distribution analysis revealed a preferential localization of euchromatin in the peripheral and intermediate regions for pre-adipocytes, contrasting with a more homogenous distribution in mature adipocytes. These findings underscore the spatial dynamics of euchromatin remodeling during adipocyte maturation, providing valuable insights into the regulatory processes governing adipogenesis. In conclusion, our multidimensional approach to visualizing and analyzing higher-order euchromatin structure within the nucleus offers a significant advancement in our understanding of nuclear organization. By revealing subnuclear interactions and spatial arrangements, this study contributes to the broader field of epigenetics and nuclear biology, opening doors for further investigations and potential applications in diverse areas of biological research. We explored the idea of creating a segmentation tool to identify the nucleus of cells in real time that facilitates a wide range of biological applications. In particular, the plugin quantifies fluorescence intensity to visualize the spatial organization of epigenetic markers or proteins relevant to dynamic cellular processes. It does so by revealing a concentric remodeling pattern within distinct areas. These concentric regions, distinguished by the structured layout of chromatin domains and/or epigenetic markers. The developed tool provides valuable insights into deciphering the spatial distribution and structural rearrangement of chromatin domains within the nucleus, which also depends on epigenetic modifications of DNA and/or histones. Our research aims to expand the field of epigenetics and provides fresh perspectives on the impact of concentric area pattern remodeling on cellular physiology and pathology.

IsoConcentraChromJ is released open source: https://github.com/dabbous94/IsoConcentraChromJ, as well as in the supporting information S1 for 2D and S3 for 3D. The repository contains one file for each of the two presented case studies 2D and 3D.

## Supporting information

S1 Fig Graphical workflow. (TIF)

S1 Script 2D IsoConcentraChromJ. (PDF)

S2 Script 3D IsoConcentraChromJ. (PDF)
