# Supplementary figures and images for: Unveiling nuclear chromatin distribution using IsoConcentraChromJ: A flourescence imaging plugin for IsoRegional and IsoVolumetric based ratios analysis
Source: PLoS One. 2024 Jul 2;19(7):e0305809. doi: 10.1371/journal.pone.0305809 (PMC11218964; doi:10.1371/journal.pone.0305809)

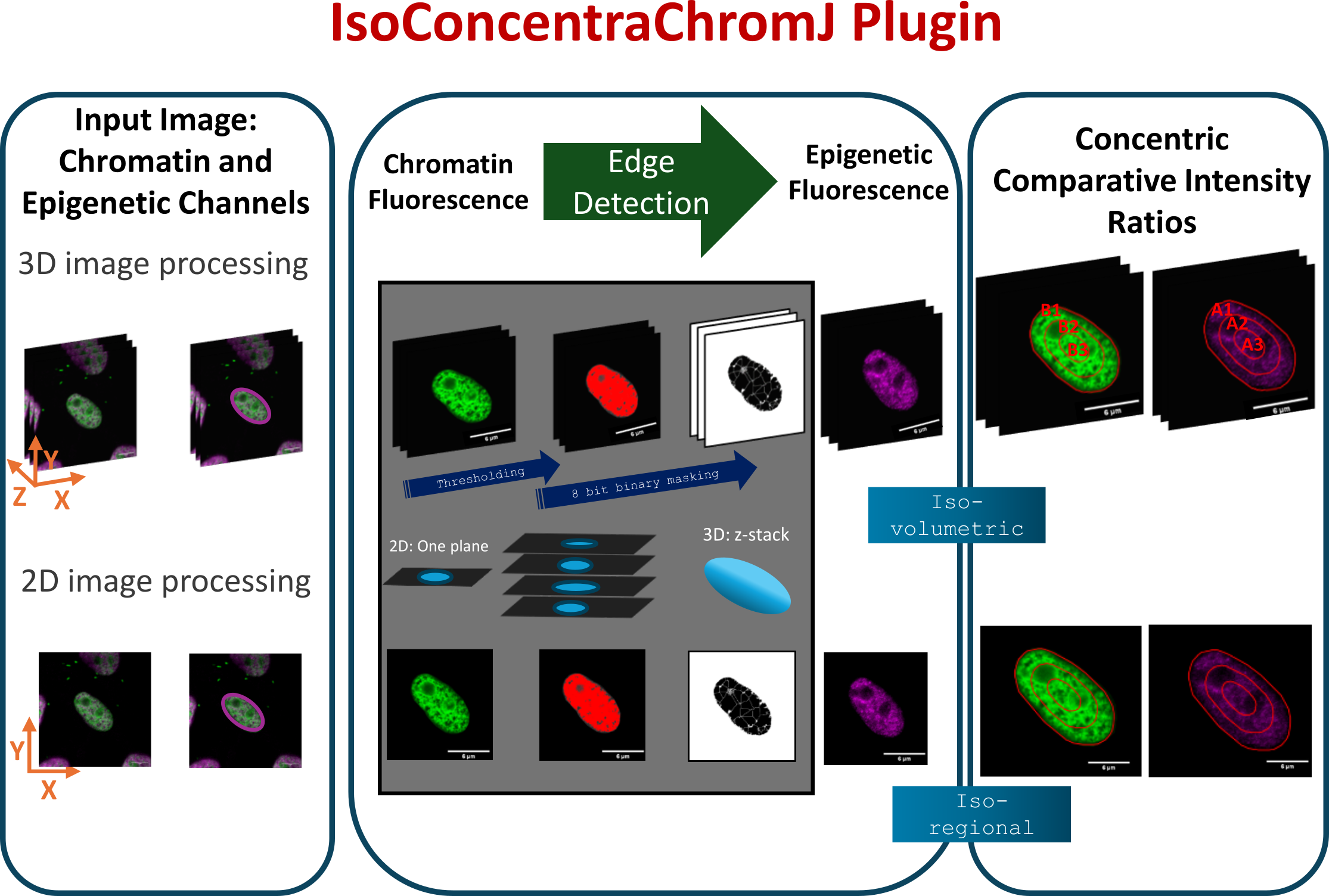

Supplement: S1 Fig — (TIF) [file pone.0305809.s001.tif]
