# Supplementary material for: Unveiling nuclear chromatin distribution using IsoConcentraChromJ: A flourescence imaging plugin for IsoRegional and IsoVolumetric based ratios analysis
Source: PLoS One. 2024 Jul 2;19(7):e0305809. doi: 10.1371/journal.pone.0305809 (PMC11218964; doi:10.1371/journal.pone.0305809)
Supplement: S1 Script — (PDF) [file pone.0305809.s002.pdf]

```
//Author: ALi Dabbous

run("Split Channels");
selectImage(1);
rename("C1");
selectImage(2);
rename("C2");
selectImage(3);
rename("C3");
selectImage(4);
rename("C4");
selectWindow("C1");
run("Duplicate...", "title=C1Duplicated");



selectWindow("C1Duplicated");
roiManager("select", 0);
run("Clear Results");
run("Measure");
mean_background=getResult("Mean");
print(" Background channel one: " + mean_background);

selectWindow("C4");
roiManager("select", 0);
run("Clear Results");
run("Measure");
mean_background1=getResult("Mean");
print( " Background channel two: " + mean_background1);
```

```
selectWindow("C1");
roiManager("select", 1);
run("Clear Outside");



selectImage("C1");
setAutoThreshold("Triangle dark");
run("Create Selection");
run("Make Inverse");
getStatistics(area, mean);
C1bg=mean;
run("Select None");
run("Subtract...", "value="+C1bg);
setAutoThreshold("Triangle dark");
run("Create Selection");
setBackgroundColor(0, 0, 0);
run("Clear Outside");
run("Enhance Contrast...", "saturated=0.1 equalize");


selectWindow("C1");
run("Select None");
run("Duplicate...", "title=thresholded1");
run("Duplicate...", "title=threshold1");
run("Threshold...");
waitForUser("Set threshold and click OK")
```

```
run("Convert to Mask");
run("Watershed");
run("Analyze Particles...", "size=5-Infinity circularity=0.00-1.00 show=Masks summarize");
rename("Mask1");
selectWindow("threshold1");
close();



selectWindow("Mask1");


run("Find Edges");


run("Create Selection");
run("Convex Hull");
getSelectionCoordinates(xpoints, ypoints);
selectWindow("C1Duplicated");
makeSelection("polygon", xpoints, ypoints);
run("Clear Results");
run("Measure");
intDen_channel1 = getResult("IntDen");
area_channel1=getResult("Area");
print( "IntDen channel one full: " + intDen_channel1);
print(" Area channel one full: " + area_channel1);
selectWindow("C4");
makeSelection("polygon", xpoints, ypoints);
run("Clear Results");
run("Measure");
intDen_channel2 = getResult("IntDen");
area_channel2=getResult("Area");
```

```
print("IntDen channel two full: "+intDen_channel2);
print("Area channel two full: "+ area_channel2 );
roiManager("Add");


sumx=0
sumy=0
for (i=0;i<lengthOf(xpoints);i++){
sumx+=xpoints[i];
sumy+=ypoints[i];
}
// Calculate the centroid of the polygon
centroid_x = sumx/ lengthOf(xpoints);
centroid_y = sumy / lengthOf(ypoints);


shrink_factor = 0.6666;
x_shrunk = newArray();
y_shrunk = newArray();
for (i = 0; i < lengthOf(xpoints); i++) {
x_shrunk[lengthOf(x_shrunk)] = (xpoints[i] - centroid_x) * shrink_factor + centroid_x;
y_shrunk[lengthOf(y_shrunk)] = (ypoints[i] - centroid_y) * shrink_factor + centroid_y;
}
// Close the shrunk polygon by repeating the first point
x_shrunk = Array.concat(x_shrunk, x_shrunk[0]);
y_shrunk = Array.concat(y_shrunk, y_shrunk[0]);



makeSelection("polygon", x_shrunk, y_shrunk);
run("Clear Results");
run("Measure");
```

```
intDen_channel2_two = getResult("IntDen");
area_channel2_two=getResult("Area");
print("IntDen channel Two medium : " +intDen_channel2_two);
print("Area channel Two medium: " +area_channel2_two );
roiManager("Add");
//

shrink_factor = 0.3333;
x_shrunk2 = newArray();
y_shrunk2 = newArray();
for (i = 0; i < lengthOf(xpoints); i++) {
x_shrunk2[lengthOf(x_shrunk2)] = (xpoints[i] - centroid_x) * shrink_factor + centroid_x;
y_shrunk2[lengthOf(y_shrunk2)] = (ypoints[i] - centroid_y) * shrink_factor + centroid_y;
}
// Close the shrunk polygon by repeating the first point
x_shrunk2 = Array.concat(x_shrunk2, x_shrunk2[0]);
y_shrunk2 = Array.concat(y_shrunk2, y_shrunk2[0]);



makeSelection("polygon", x_shrunk2, y_shrunk2);
run("Clear Results");
run("Measure");
intDen_channel2_three = getResult("IntDen");
area_channel2_three=getResult("Area");
print("IntDen channel Two small: " +intDen_channel2_three);
print("Area channel Two small: " +area_channel2_three );
roiManager("Add");

////
```

```
selectWindow("C1Duplicated");
makeSelection("polygon", x_shrunk, y_shrunk);
run("Clear Results");
run("Measure");
intDen_channel1_two = getResult("IntDen");
area_channel1_two=getResult("Area");
print("IntDen channel one medium: " +intDen_channel1_two);
print("Area channel one medium: " +area_channel1_two );


makeSelection("polygon", x_shrunk2, y_shrunk2);
run("Clear Results");
run("Measure");
intDen_channel1_three = getResult("IntDen");
area_channel1_three=getResult("Area");
print("IntDen channel one small: " +intDen_channel1_three );
print("Area channel one small: " +area_channel1_three );



////Normalization
volume_c11_normalized=(intDen_channel1) - (mean_background*area_channel1);
volume_c12_normalized=(intDen_channel1_two) - (mean_background*area_channel1_two);
volume_c13_normalized=(intDen_channel1_three)-(mean_background*area_channel1_three);
volume_c21_normalized=(intDen_channel2) - (mean_background1*area_channel2);
volume_c22_normalized=(intDen_channel2_two) - (mean_background1*area_channel2_two);
volume_c23_normalized=(intDen_channel2_three)-(mean_background1*area_channel2_three);

volume_channel_one=(intDen_channel1/area_channel1) - (mean_background*area_channel1);
```

```
volume_channel_two=(intDen_channel2/area_channel2) - (mean_background1*area_channel2);

//// Ratio calculation

ratio_one=volume_c21_normalized/volume_c11_normalized ;
ratio_two=(volume_c21_normalized-volume_c22_normalized)/volume_c11_normalized;
ratio_three=(volume_c21_normalized-volume_c22_normalized)/volume_c21_normalized;
ratio_four=(volume_c22_normalized-volume_c23_normalized)/volume_c11_normalized;
ratio_five=(volume_c22_normalized-volume_c23_normalized)/volume_c21_normalized;
ratio_six=(volume_c23_normalized)/volume_c11_normalized;
ratio_seven=(volume_c23_normalized)/volume_c21_normalized;

ratio_eight=(volume_c11_normalized-volume_c12_normalized)/volume_c11_normalized;
ratio_nine=(volume_c12_normalized-volume_c13_normalized)/volume_c11_normalized;
ratio_ten=(volume_c13_normalized)/volume_c11_normalized;



print("A/B: " + ratio_one);
print("A1/B: " +ratio_two);
print("A1/A: " +ratio_three);
print("A2/B: " +ratio_four);
print("A2/A: " +ratio_five);
print("A3/B: " +ratio_six);
print("A3/A: " +ratio_seven);
print("B1/B: " +ratio_eight);
print("B2/B: " +ratio_nine);
print("B3/B: " +ratio_ten);
```
