# Supplementary material for: Unveiling nuclear chromatin distribution using IsoConcentraChromJ: A flourescence imaging plugin for IsoRegional and IsoVolumetric based ratios analysis
Source: PLoS One. 2024 Jul 2;19(7):e0305809. doi: 10.1371/journal.pone.0305809 (PMC11218964; doi:10.1371/journal.pone.0305809)
Supplement: S2 Script — (PDF) [file pone.0305809.s003.pdf]

```
// Author: Ali Dabbous

run("Split Channels");
selectImage(1);
rename("C1");
selectImage(2);
rename("C2");
selectImage(3);
rename("C3");
selectWindow("C1");
run("Duplicate...", "duplicate slices=nSlices()");
rename("C1Duplicated");




selectWindow("C1Duplicated");
roiManager("select", 0);
run("Clear Results");
for(n=1;n <= nSlices();n++){
setSlice(n);
run("Measure");
}
total_mean_channel1=0;
for (i = 0; i < nResults(); i++) {
   total_mean_channel1+ = getResult("Mean", i);
}
getVoxelSize(width, height, depth, unit);
```

```
total_mean_channel1=total_mean_channel1/nSlices();
volume_background=total_mean_channel1*depth;
print("background channel one"+ volume_background);


roiManager("select", 1);
run("Clear Outside");


selectWindow("C2");
roiManager("select", 0);
run("Clear Results");
for(n=1;n <= nSlices();n++){
setSlice(n);
run("Measure");
}
total_mean_channel2=0;
for (i = 0; i < nResults(); i++) {
   total_mean_channel2+ = getResult("Mean", i);
}
getVoxelSize(width, height, depth, unit);
total_mean_channel2=total_mean_channel2/nSlices();
volume_background2=total_mean_channel2*depth;
print("background channel two" + volume_background2);




selectWindow("C1");
roiManager("select", 1);
run("Clear Outside");
```

```
selectImage("C1");
setAutoThreshold("Triangle dark");
run("Create Selection");
run("Make Inverse");
getStatistics(area, mean);
C1bg=mean;
run("Select None");
run("Subtract...", "value="+C1bg);
setAutoThreshold("Triangle dark");
run("Create Selection");
setBackgroundColor(0, 0, 0);
run("Clear Outside");
run("Enhance Contrast...", "saturated=0.1 equalize");



selectWindow("C1");
run("Select None");
run("Duplicate...", "title=thresholded1");
run("Duplicate...", "title=threshold1");
run("Threshold...");
waitForUser("Set threshold and click OK")
run("Convert to Mask");
run("Watershed");
run("Analyze Particles...", "size=5-Infinity circularity=0.00-1.00 show=Masks summarize");
rename("Mask1");
selectWindow("threshold1");
```

```
close();

selectWindow("Mask1");
run("Find Edges");
run("Create Selection");
run("Convex Hull");
getSelectionCoordinates(xpoints, ypoints);
selectWindow("C1Duplicated");
makeSelection("polygon", xpoints, ypoints);
run("Clear Results");
for(n=1;n < nSlices();n++){
setSlice(n);
run("Measure");
}
```

```
total_intDen=0;
total_area=0;
for (i = 0; i < nResults(); i++) {
    total_intDen+ = getResult("IntDen", i);
    total_area+ = getResult("Area", i);
}
getVoxelSize(width, height, depth, unit);
volume_channel_one=total_intDen*depth;
area_channel_one=total_area*depth;
print("Intden channel one full" +volume_channel_one);
print(" Area channel one full: "+area_channel_one);
selectWindow("C2");
makeSelection("polygon", xpoints, ypoints);
run("Clear Results");
for(n=1;n<nSlices();n++){
setSlice(n);
run("Measure");
}
total_intDen=0;
total_area=0;
for (i = 0; i < nResults(); i++) {
    total_intDen+ = getResult("IntDen", i);
    total_area+ = getResult("Area", i);
}
getVoxelSize(width, height, depth, unit);
volume_channel_two_one=total_intDen*depth;
area_channel_two_one=total_area*depth;
print("IntDen channel two full: "+volume_channel_two_one );
print("Area channel two full: "+area_channel_two_one );
```

```
roiManager("Add");


sumx=0
sumy=0
for (i=0;i<lengthOf(xpoints);i++){
sumx+=xpoints[i];
sumy+=ypoints[i];
}
// Calculate the centroid of the polygon
centroid_x = sumx/ lengthOf(xpoints);
centroid_y = sumy / lengthOf(ypoints);
// Shrink the polygon by 30% while keeping the centroid fixed
shrink_factor = 0.6666;
x_shrunk = newArray();
y_shrunk = newArray();
for (i = 0; i < lengthOf(xpoints); i++) {
x_shrunk[lengthOf(x_shrunk)] = (xpoints[i] - centroid_x) * shrink_factor + centroid_x;
y_shrunk[lengthOf(y_shrunk)] = (ypoints[i] - centroid_y) * shrink_factor + centroid_y;
}
// Close the shrunk polygon by repeating the first point
x_shrunk = Array.concat(x_shrunk, x_shrunk[0]);
y_shrunk = Array.concat(y_shrunk, y_shrunk[0]);



makeSelection("polygon", x_shrunk, y_shrunk);
run("Clear Results");
for(n=1;n<nSlices();n++){
setSlice(n);
run("Measure");
```

```
}
total_intDen=0;
total_area=0;
for (i = 0; i < nResults(); i++) {
   total_intDen+ = getResult("IntDen", i);
   total_area+ = getResult("Area", i);
}
getVoxelSize(width, height, depth, unit);
volume_channel_two_two=total_intDen*depth;
area_channel_two_two=total_area*depth;
print("IntDen channel Two medium : " +volume_channel_two_two );
print("Area channel Two medium: " +area_channel_two_two );
roiManager("Add");

// Shrink the polygon by 60% while keeping the centroid fixed
shrink_factor = 0.3333;
x_shrunk2 = newArray();
y_shrunk2 = newArray();
for (i = 0; i < lengthOf(xpoints); i++) {
x_shrunk2[lengthOf(x_shrunk2)] = (xpoints[i] - centroid_x) * shrink_factor + centroid_x;
y_shrunk2[lengthOf(y_shrunk2)] = (ypoints[i] - centroid_y) * shrink_factor + centroid_y;
}
// Close the shrunk polygon by repeating the first point
x_shrunk2 = Array.concat(x_shrunk2, x_shrunk2[0]);
y_shrunk2 = Array.concat(y_shrunk2, y_shrunk2[0]);


makeSelection("polygon", x_shrunk2, y_shrunk2);
run("Clear Results");
```

```
for(n=1;n<nSlices();n++){
setSlice(n);
run("Measure");
}
total_intDen=0;
total_area=0;
for (i = 0; i < nResults(); i++) {
    total_intDen+ = getResult("IntDen", i);
    total_area+ = getResult("Area", i);
}
getVoxelSize(width, height, depth, unit);
volume_channel_two_three=total_intDen*depth;
area_channel_two_three=total_area*depth;
print("IntDen channel Two small: " +volume_channel_two_three);
print("Area channel Two small: " + area_channel_two_three);
roiManager("Add");


selectWindow("C1Duplicated");
makeSelection("polygon", x_shrunk, y_shrunk);
run("Clear Results");
for(n=1;n<nSlices();n++){
setSlice(n);
run("Measure");
}
total_intDen=0;
total_area=0;
for (i = 0; i < nResults(); i++) {
    total_intDen+ = getResult("IntDen", i);
```

```
    total_area+ = getResult("Area", i);
}
getVoxelSize(width, height, depth, unit);
volume_channel_one_two=total_intDen*depth;
area_channel_one_two=total_area*depth;
print("IntDen channel one medium : " +volume_channel_one_two );
print("Area channel one medium: " +area_channel_one_two );



makeSelection("polygon", x_shrunk2, y_shrunk2);
run("Clear Results");
for(n=1;n<nSlices();n++){
setSlice(n);
run("Measure");
}
total_intDen=0;
total_area=0;
for (i = 0; i < nResults(); i++) {
    total_intDen+ = getResult("IntDen", i);
    total_area+ = getResult("Area", i);
}
getVoxelSize(width, height, depth, unit);
volume_channel_one_three=total_intDen*depth;
area_channel_one_three=total_area*depth;
print("IntDen channel one small: " +volume_channel_one_three);
print("Area channel one small: " + area_channel_one_three);
```

```
////Normalization

volume_c1_normalized=volume_channel_one - (volume_background*area_channel_one);

volume_c12_normalized=volume_channel_one_two - (volume_background*area_channel_one_two);

volume_c13_normalized=volume_channel_one_three -
(volume_background*area_channel_one_three);

volume_c21_normalized=volume_channel_two_one - (volume_background2*area_channel_two_one);

volume_c22_normalized=volume_channel_two_two - (volume_background2*area_channel_two_two);

volume_c23_normalized=volume_channel_two_three-
(volume_background2*area_channel_two_three);



//// Ratio calculation


ratio_one=volume_c21_normalized/volume_c1_normalized ;

ratio_two=(volume_c21_normalized-volume_c22_normalized)/volume_c1_normalized;

ratio_three=(volume_c21_normalized-volume_c22_normalized)/volume_c21_normalized;

ratio_four=(volume_c22_normalized-volume_c23_normalized)/volume_c1_normalized;

ratio_five=(volume_c22_normalized-volume_c23_normalized)/volume_c21_normalized;

ratio_six=(volume_c23_normalized)/volume_c1_normalized;

ratio_seven=(volume_c23_normalized)/volume_c21_normalized;


ratio_eight=(volume_c1_normalized-volume_c12_normalized)/volume_c1_normalized;

ratio_nine=(volume_c12_normalized-volume_c13_normalized)/volume_c1_normalized;

ratio_ten=(volume_c13_normalized)/volume_c1_normalized;



print("A/B: " + ratio_one);

print("A1/B: " +ratio_two);
```

```
print("A1/A: " +ratio_three);

print("A2/B: " +ratio_four);

print("A2/A: " +ratio_five);

print("A3/B: " +ratio_six);

print("A3/A: " +ratio_seven);

print("B1/B: " +ratio_eight);

print("B2/B: " +ratio_nine);

print("B3/B: " +ratio_ten);
```
